# Development and Preliminary Validation of a Screener for Digital Health Readiness

**DOI:** 10.1001/jamanetworkopen.2024.32718

**Published:** 2024-09-10

**Authors:** Kristin L. Rising, Amanda Guth, Alexzandra T. Gentsch, Karla Martin Gonzalez, Richard Hass, Lindsey Shughart, Serena Gelfer, Megan McVane, Brooke Worster, Amy E. Leader

**Affiliations:** 1 Center for Connected Care, Thomas Jefferson University, Philadelphia, Pennsylvania; 2 Department of Emergency Medicine, Thomas Jefferson University, Philadelphia, Pennsylvania; 3 College of Population Health, Thomas Jefferson University, Philadelphia, Pennsylvania; 4 Department of Geriatrics, Edward Hines Jr Veterans Administration Hospital, Hines, Illinois; 5 Division of Supportive Medicine, Department of Medical Oncology, Thomas Jefferson University, Philadelphia, Pennsylvania; 6 Sidney Kimmel Cancer Center, Thomas Jefferson University, Philadelphia, Pennsylvania; 7 Division of Population Science, Department of Medical Oncology, Thomas Jefferson University, Philadelphia, Pennsylvania

## Abstract

**Importance:**

While telehealth use has grown, patient uptake is variable, which has the potential to increase health disparities. Identifying and understanding individuals’ barriers to digital health readiness can help health systems efficiently deploy resources to provide personalized patient-centered support.

**Objective:**

To develop and validate an instrument to evaluate digital health readiness to identify and quantify barriers to digital readiness.

**Design, Setting, and Participants:**

In this qualitative mixed-methods study conducted from April 26, 2022, to June 8, 2023, the instrument was created in 4 phases. Patients and health care professionals were interviewed to explore barriers to telehealth use, scale items were developed for the screener, cognitive interviews were conducted to refine scale items, and psychometric properties of the screener were evaluated. The study was conducted in an urban, multisite academic health system and the surrounding community. Participants were older than 18 years, English-speaking, and able to provide informed consent. Professionals worked within the Jefferson Health system and were involved in telehealth services.

**Exposure:**

Participants completed a semistructured interview (duration: 6-19 minutes), a cognitive interview (duration: 13-137 minutes), or the draft screener survey (duration: 5-10 minutes).

**Main Outcomes and Measures:**

Development and validation of a screener for digital health readiness.

**Results:**

Of 519 patients approached, 19 were ineligible, 122 declined, and 11 were excluded from analysis, resulting in inclusion of 367 participants (32 patient interviews, 16 professional interviews, 15 cognitive interviews, 304 psychometric survey testing). All 16 professionals who were approached participated. Most patient participants were Black (46.7%) or White (37.9%), male (56.4%), and had a high school degree or some college (49.6%); mean (SD) age was 45 (23) years for participants in cognitive interviews, 53 (18) years for survey respondents, and 57 (14) years for patient interviews. The structured interviews uncovered 21 concepts, leading to 48 items that were refined through cognitive interviews. Psychometric analyses of the 29 items that emerged from the cognitive interviews resulted in a final screener with 24 items across 2 factors: technical readiness (18 items; factor loading range, 0.488-0.968) and quality-of-care concerns (6 items; factor loading range, 0.619-0.942).

**Conclusions and Relevance:**

In this qualitative study of digital health readiness, the findings suggest that the screener items may be valid to assess the complexity of factors influencing digital health uptake and highlight several areas for potential intervention.

## Key Points

**Question:**

Does a newly developed digital tool show validity for use by patients in health care engagement?

**Findings:**

This qualitative study of a screener for digital health readiness, which measures technical readiness and quality-of-care concerns, included data from 367 participants. Forty-eight scale items were developed, 29 of which were examined for psychometric purposes: the final screener consists of 24 items that comprise 2 factors, 1 with 18 items (technical readiness) and the other with 6 items (quality-of-care concerns), with psychometric analyses yielding early construct validity.

**Meaning:**

Preliminary testing of the digital tool suggests the validity and utility of this tool to barriers individuals may face when accessing, understanding, or using digital health tools that provide personalized patient-centered support.

## Teaser

This qualitative study develops and performs preliminary validity analysis of a tool to measure patients’ digital health readiness.

## Introduction

The COVID-19 pandemic facilitated unprecedented growth in telehealth use across the US. While the rapid uptake of virtual care helped ensure care continuity while enabling social distancing,^1^ it required resources and skills that are not distributed equally among health care consumers. As has been reported,^2,3^ simply having a device, such as a smartphone or tablet, does not mean that someone can or will use it to access their health information or engage in other digital health services.^4^ Studies exploring health literacy, digital health literacy, and barriers to use of telehealth have identified a range of important barriers to telehealth uptake that go beyond simply access to and knowledge in use of devices. These barriers include factors such as trust, acceptance, and understanding the relevance of telehealth as a means of receiving care, and they have been reported to independently be associated with lower uptake of telehealth.^4,5,6,7,8^

Expanding on the concept of digital readiness,^4^ our team adopted the term *digital health readiness* to represent the overarching concept of people’s ability and comfort in using digital tools for health care engagement, inclusive of the many factors that extend beyond digital health literacy. Understanding each patient’s digital health readiness can help inform health systems on how to most efficiently and effectively deploy digital health readiness interventions across their population served. Yet operationalizing this concept requires a means of quantifying digital health readiness.

While there are existing tools to assess digital health literacy, including a recently developed Digital Health Readiness Questionnaire, none of these also incorporates other important readiness factors, such as people’s trust in digital health services.^5,9,10^ A standardized approach to measure digital health readiness, with a focus on identifying each individual’s specific barriers to readiness, is needed to inform delivery of tailored interventions focused on addressing these barriers. To that end, the objective of this work was to develop and conduct preliminary validation of a screener for digital health readiness.

## Methods

### Design and Setting

This was a qualitative mixed-methods study conducted at Thomas Jefferson University in Philadelphia, Pennsylvania, from April 26, 2022, to June 8, 2023. Thomas Jefferson University and Jefferson Health comprise an 18-hospital academic health system that spans 9 counties in 2 states and is the largest health system in the Philadelphia region. The catchment area covers more than 5.5 million residents and includes counties that are home to some of the most socioeconomically diverse populations in the Philadelphia area and the US. Poverty rates range from 21.7% in Philadelphia County, Pennsylvania, to 5.3% in Chester County, Pennsylvania, with an average at 11.7%. All study activities were approved by the Thomas Jefferson University Institutional Review Board. Qualitative results are reported based on the Standards for Reporting Qualitative Research (SRQR) reporting guideline.^11^ None of the researchers had a preexisting relationship with the patient study participants, but some of us (K.L.R., B.W., and A.E.L.) had previous working relationships with some of the health care professional participants. Data were deidentifed. Patients provided verbal informed consent.

### Interviews

For the first stage of this project, we conducted individual semistructured interviews (duration: 6-19 minutes) with a diverse sample of patients and professionals to ensure comprehensive concept elicitation of digital health readiness. The patient interview guide was designed to explore patient-important domains of digital health readiness, such as the ability to locate credible health information online, find and download a health app, log into a patient portal, or use telehealth for a clinical visit, as well as explore themes associated with trust of digital health information (eAppendix 1 in Supplement 1). Professional interviews focused on exploring what professionals believe are important digital skills for patients to manage their health and wellness and the benefits and burdens associated with digital engagement with patients (eAppendix 2 in Supplement 1). Guides were informally pilot tested with members of the team and were iteratively refined based on results from the first interviews.

All individuals who were older than 18 years, English-speaking, and able to provide informed consent were eligible to participate in the patient interviews. They were recruited by research team members at various Jefferson Health sites and community events. Inclusion criteria for the professional interviews included being a clinician, telehealth support staff, or health informatics professional who worked at Jefferson Health and was involved with direct provision of or supporting provision of telehealth services to Jefferson Health patients. All health care professionals were recruited through email.

Individuals participating in the patient interviews completed a demographic questionnaire. Interviews were conducted by team members (A.G., A.T.G., and K.M.G.) who were overseen by other members of the team with extensive qualitative expertise (K.L.R. and A.E.L.). Interviews were continued until thematic saturation was achieved. Interviews were audio recorded and transcribed by a professional transcription agency, with identifying information removed.

Transcripts were loaded into NVivo, version 14 (QSR International),^12^ for analysis. We applied a conventional content analysis approach to identify themes related to digital health readiness with the purpose of extracting relevant data for use in item development of the tool. The entire research team first reviewed a subset of transcripts to develop an initial codebook to capture items that could be considered a concept of digital readiness. Two team members (K.M.G. and L.S.) then applied the codebook to all transcripts, with percent agreement and κ coefficient regularly reviewed throughout the coding process. An additional team member (A.T.G.) met regularly with the coders throughout the coding process to compare coding, resolve discrepancies, and refine the codebook as needed to ensure broad inclusion of concepts suggesting digital health readiness. Ultimately, 36% of the transcripts were double-coded.^13,14^

### Item Generation

In the second stage, we used concepts identified within the interviews to develop an initial set of items to assess digital health readiness barriers. To do so, 2 team members (A.T.G. and S.G.) reviewed coded data from the transcripts to develop an initial list of concepts. The team generated a list of items to be inclusive of all the relevant content.

### Cognitive Interviews

In the third stage, we conducted cognitive interviews (duration: 13-137 minutes) with a convenience sample of patients to refine the draft tool. Cognitive interviews are a qualitative research method used to enhance the accuracy and reliability of survey instruments.^15^ The technique involves detailed in-depth interviews with respondents to understand how they interpret questions and to identify potential issues with question wording, structure, or response options. Interviews assessed the clarity of items and content validity of the tool as a whole. Eligible patients were adults (aged ≥18 years), English-speaking, able to provide informed consent, and receiving care at either the Thomas Jefferson University Hospital emergency department or the Jefferson Sidney Kimmel Cancer Center infusion center. These 2 sites were selected to allow for increased diversity of patients enrolled.

Cognitive interviews were completed by 2 members of the research team (K.M.G. and S.G.). All participants provided verbal informed consent and completed a demographic characteristic questionnaire at the time of the cognitive interview. During the interview, participants were asked to provide an answer to each item within the draft tool (duration: 5-10 minutes). They were then also asked questions about each item to assess the item’s understandability, acceptability, and appropriateness.^15^ At the end of the individual item review, participants were asked to identify any items that should be eliminated and whether any other items needed to be added. The tool was iteratively modified based on interview responses, and interviews were continued until no further changes to the tool were needed. A detailed audit trail was kept of all decisions.

### Statistical Analysis

For our final stage, we conducted psychometric analysis of the tool among a large sample of patients to inform further refinement of the items and assess initial screener structure and performance. Participant inclusion criteria included age 18 years or older, English-speaking, and able to provide informed consent. We purposefully sampled patients through an initial screening process who were older, of a minority race and ethnicity, and/or had low educational attainment, as prior studies have documented lower digital literacy among these populations, and we wanted to ensure inclusion of individuals with a broad range of digital health readiness.

Psychometric analyses began with exploratory item factor analysis using methods developed for binary items.^16,17^ The primary goal was to determine whether the item set was unidimensional, so multifactor solutions were compared with the unidimensional solutions via a likelihood ratio χ^2^ test. A final multifactor solution was chosen based on interpretability. Concurrent and discriminant validity analyses were performed using nonparametric methods with composite scores as outcomes and the following covariates: self-reported health literacy,^18^ participant age, educational attainment, and race and ethnicity. Nonparametric tests were used as the scores showed substantial departure from normality. Spearman rank-order correlations were computed between scores on the tool and numeric covariates, and Kruskal-Wallis tests were run to examine differences in the distribution of scores across categorical variable levels. All statistical analyses were performed in R, version 4.3.2 (R Foundation for Statistical Computing). The 2-sided significance threshold was *P* < .05; all testing was unpaired.

## Results

### Participants

Of 519 patients approached, 19 were ineligible and 122 declined. Of 378 individuals enrolled, 11 were excluded from analysis, resulting in data on 367 individuals (32 patient interviews, 16 clinician and telehealth support interviews, 15 patient cognitive interviews, and 304 patient surveys for psychometric testing), with most patients self-reported as Black (46.7%) or White (37.9%) and male (56.4%), with a high school degree or some college-level education (49.6%); mean (SD) age was 45 (23) years for participants in cognitive interviews, 53 (18) years for survey respondents, and 57 (14) years for patient interviews (Table 1 and Table 2).

**Table 1.** Patient Participant Demographic Characteristics

Characteristic	No. (%)		
	Interviews		Surveys (n = 304)
	Patient (n = 32)	Cognitive (n = 15)	
Gender			
Female	16 (50.0)	7 (46.7)	125 (41.1)
Male	16 (50.0)	8 (53.3)	174 (57.2)
Nonbinary	0	0	5 (1.6)
Age, mean (SD), y	57 (14)	45 (23)	53 (18)
Race^a^			
American Indian or Alaska Native	0	0	0
Asian	1 (3.1)	0	10 (3.2)
Black	17 (53.1)	7 (46.7)	140 (46.1)
Middle Eastern	1 (3.1)	0	22 (7.2)
Native Hawaiian or Other Pacific Islander	1 (3.1)	0	2 (0.6)
White	8 (25.0)	7 (46.7)	118 (38.8)
Other (not specified)^b^	0	1 (6.7)	0
≥2 Races	0	0	12 (3.9)
Missing	4 (12.5)	0	0
Ethnicity^c^			
Hispanic or Latino	5 (15.6)	0	36 (11.8)
Non-Hispanic	27 (84.4)	15 (100.0)	267 (87.8)
Declined to answer	0	0	1 (0.3)
Education level			
Less than high school	5 (15.6)	1 (6.7)	25 (8.2)
High school graduate, GED, some college	20 (62.5)	7 (46.7)	147 (48.4)
College degree	4 (12.5)	6 (40.0)	89 (29.3)
Postgraduate degree	3 (9.4)	1 (6.7)	43 (14.1)
Annual household income, $			
<10 000	6 (18.8)	0	NA
10 000-24 999	7 (21.9)	4 (26.7)	NA
25 000-49 999	10 (31.3)	4 (26.7)	NA
50 000-99 999	3 (9.4)	3 (20.0)	NA
>100 000	2 (6.3)	1 (6.7)	NA
Unsure	1 (3.1)	1 (6.7)	NA
Declined to answer	3 (9.4)	2 (13.3)	NA
Health literacy score, mean (SD)^14^^,^^d^	NA	NA	17 (3.3)
Insurance			
Medicaid/Medicare	NA	NA	163 (53.6)
Private	NA	NA	129 (42.4)
Uninsured	NA	NA	7 (2.3)
Missing	NA	NA	5 (1.6)
Food insecurity			
Yes	NA	NA	60 (19.7)
No	NA	NA	243 (79.9)
Missing	NA	NA	1 (0.3)
Disability			
Yes	NA	NA	90 (29.6)
No	NA	NA	214 (70.4)

Abbreviations: GED, general educational development; NA, not available.

^a^ Race was identified from self-report during data collection.

^b^ Ethnicity was identified from self-report during data collection.

^c^ No further breakdown of this classification is available.

^d^ Health literacy score rating: limited, 4 to 12; marginal, 13 to 16; adequate, 17 to 20.

**Table 2.** Clinician and Support Staff Demographic Characteristics With 16 Interviews

Characteristic	No. (%)
Gender	
Female	11 (68.8)
Male	5 (31.3)
Nonbinary	0
Professional area of specialty	
Clinician primary care	6 (37.5)
Clinician specialist	2 (12.5)
Health information management and clinical documentation improvement	6 (37.5)
Social worker	2 (12.5)
Race^a^	
American Indian or Alaska Native	0
Asian	3 (18.8)
Black	2 (12.5)
Middle Eastern	0
Native Hawaiian or Other Pacific Islander	0
White	11 (68.8)
Ethnicity^b^	
Hispanic or Latino	1 (6.3)
Non-Hispanic	15 (93.8)

^a^ Race was identified from self-report during data collection.

^b^ Ethnicity was identified from self-report during data collection.

### Item Development

Review of the interview data resulted in identification of 21 concepts from which the team developed a list of 48 items. For many items, it was initially unclear whether it was preferred to structure the question as a yes or no choice or a Likert scale, so many items were generated with both structures, allowing the final decision about the optimal structure to be made during cognitive interviews.

### Cognitive Interviews

Cognitive interviews resulted in revised wording of 15 items, revised wording of scale responses for 7 items, reversed scale responses for 7 items, adding an additional response option to 2 items, adding 4 items, and removing 23 items. In addition, a prompt was added to one section of the screener to increase clarity.

### Psychometric Analysis

A total of 29 items were examined for psychometric purposes. Eight items had multiple response categories that were collapsed into binary yes or no options to aid in the scoring of the tool and the use of exploratory item factor analysis, and 7 items were reverse scored before factor analysis. Table 3 contains the text for each item, the percentage of participants answering yes (or no for reverse-scored items), and factor loadings from a 2-factor solution (with oblique rotation). The 2-factor structure was the most interpretable solution, with most of the items (n = 18; items 1-7, 9-16, 18, 20, and 29) loading together on the factor subsequently named technical readiness factor (eg, “Do you feel comfortable accessing the internet?”). Four items (items 22-24 and 28) loaded strongly together on the second factor, named quality-of-care concerns factor (eg, “Are you concerned that you won’t get high-quality care on a telehealth video visit?”). Items 26 and 27 (privacy information and security) loaded twice as high on the quality-of-care concerns factor and were retained because they were interpreted to be important to that factor. The remaining 5 items (8, 17, 19, 21, and 25) did not exhibit a simple structure (ie, had high or low loadings on both factors) and were eliminated. The interfactor correlation was small (*r* = 0.079).

**Table 3.** Factor Loading Scores of Initial 29 Digital Health Readiness Items Based on a 2-Factor Solution

Screening item	Factor loading		Yes (no for reverse-scored items), %
	Technical readiness	Quality-of-care concerns	
1. Have you ever completed a telehealth video visit with a provider?	0.548^a^	−0.050	63
2. Are you confident in your technical ability to do a telehealth video visit now or in the future?	0.722^a^	0.022	87
3. Do you have access to the internet?	0.923^a^	−0.056	96
4. Do you have a place where you are comfortable talking about your health needs?	0.577^a^	0.057	96
5. Do you know what to do when you have issues with sound or picture quality for video calls?	0.679^a^	−0.019	69
6. Do you have access to a phone with a camera or computer with a camera?	0.756^a^	0.159	97
7. Some patients do not participate in telehealth video visits because it takes up too many data minutes. Are you willing to use your data minutes for a telehealth video visit?	0.488^a^	0.106	81
8. Do you have any physical challenges that limit your use of technology?^b^^,^^c^	0.414	0.217	89
9. Do you feel confident using a computer?	0.839^a^	−0.014	87
10. Do you feel comfortable accessing the internet?	0.968^a^	−0.057	92
11. Do you feel confident accessing the internet?	0.571^a^	0.162	99
12. Can you find health information on the internet?	0.827^a^	−0.011	95
13. Are you able to use email?	0.951^a^	0.028	92
14. Do you use email at least every week?	0.897^a^	−0.154	81
15. Can you download and install new apps when needed?	0.851^a^	0.133	89
16. Do you know how to create a new username and password for a new account?	0.888^a^	−0.085	87
17. Do you have problems remembering log in info (user name and/or password)?^b^^,^^c^	0.176	0.181	55
18. Are you able to reset your password for apps or online accounts when needed?	0.877^a^	0.002	86
19. Do you have someone who can help you use technology for your health care if you need it?^c^	0.251	0.232	87
20. Do you know what a patient portal is?	0.783^a^	−0.113	83
21. If you use a patient portal, are you able to easily find what you are looking for?^c^	0.436	0.273	88
22. Are you concerned that you won’t get high-quality care on a telehealth video visit?^b^	−0.029	0.828^a^	75
23. Are you concerned that your doctor won’t spend enough time with you on a telehealth video visit?^b^	−0.097	0.942^a^	77
24. Are you concerned that you won’t have a personal connection with a doctor during a telehealth video visit?^b^	−0.121	0.903^a^	70
25. Do you feel comfortable talking about private health information on a video call?^c^	0.391	0.289	82
26. Are you concerned about the privacy of your information when using technology for your health care?^b^	0.322	0.651^a^	65
27. Are you concerned about security of your personal information when using a patient portal?^b^	0.368	0.619^a^	65
28. Have you had a bad experience with telehealth that makes you not interested in using telehealth again?^b^	−0.007	0.665^a^	95
29. Would you be willing to use technology in new ways if you were given help or instructions?	0.532^a^	0.116	94

^a^ Simple structure and primary factor loading.

^b^ Reverse scored.

^c^ Item removed from the final tool.

Composite scores for the technical readiness subscale were computed by summing across items 1 to 7, 9 to 16, 18, 20, and 30. Composite scores for the quality-of-care concerns subscale were computed by summing across items 22 to 24 and 26 to 28, all reverse scored. Table 4 contains descriptive statistics for both subscales along with their Spearman rank-order correlations with participant age and health literacy (*r* = 0.25; 95% CI, 0.14-0.35). Technical readiness was heavily skewed with 50% of participants scoring 17 or 18 (maximum of 18), although it showed a significant negative correlation with age (*r* = −0.28; 95% CI, −0.38 to −0.17) and a positive correlation with health literacy (*r* = 0.25; 95% CI, 0.14-0.35), as expected. Similarly, the quality-of-care concerns item was skewed, with most participants indicating few concerns (ie, high readiness). A positive but smaller correlation was found between quality-of-care concerns and health literacy (*r* = 0.23; 95% CI, 0.12-0.33) but not between quality-of-care concerns and age.

**Table 4.** Descriptive Statistics of Digital Health Readiness Subscales

Screening item factor	Descriptive statistic						
	Mean (SD)	SEM	Median (range)	Skew	Measure (95% CI)		
					α Level	Correlation with age	Correlation with health literacy
Technical readiness	15.58 (3.44)	0.02	17 (1 to 18)	−2.08	0.73 (0.68 to 0.77)	−0.28 (−0.38 to −0.17)	0.25 (0.14 to 0.35)
Quality-of-care concerns	4.47 (1.71)	0.01	5 (0 to 6)	−0.86	0.75 (0.71 to 0.80)	0.10 (−0.02 to 0.21)	0.23 (0.12 to 0.33)

A Kruskal-Wallis test showed that there was statistically distinct variation in distributions of technical readiness scale scores among educational attainment categories (χ^2^_3_ = 39.13; *P* < .001). There was a nearly linear increase in the median technical readiness score as educational attainment increased across the 4 levels. Quality-of-care concerns scores did not vary significantly with educational level (χ^2^_3_ = 1.71; *P* = .64), as the median scores remained close to 6 across all 4 groups (Figure).

**Figure.**
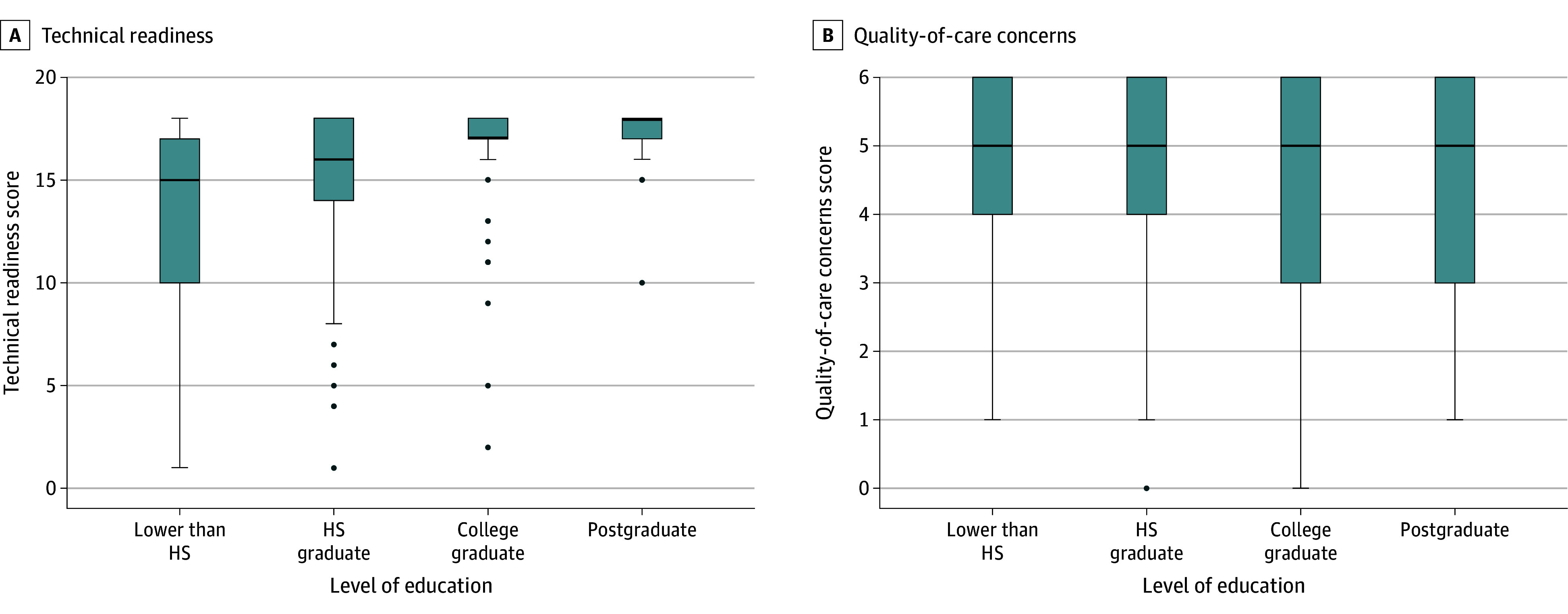
Technical Readiness and Quality-of-Care Concerns Scores Across the 4 Levels of Patient Education The bold horizontal line is the median score per group, boxes represent the middle 50% of the data, and lines extend either to the minimum or maximum score or to 1.5 times the IQR. Data more extreme than the whiskers are plotted individually as outliers. HS indicates high school.

## Discussion

In this qualitative study, we describe the process of developing the digital screening tool, which, to our knowledge, is the first one designed to assess factors beyond immediate digital skills and literacy that may affect an individual’s digital health readiness. The tool was developed with rigorous methods, including comprehensive concept elicitation across both patients and health care professionals, refinement of the tool through extensive patient feedback, and psychometric testing. Similarly, the tool was developed with a lens toward health equity, allowing the voices of patients who experience barriers in technology use to lay the foundation of the items in the tool. Results suggest that the tool comprises 2 subscales, each of which has good initial evidence for validity in measuring digital health readiness.

The psychometric analyses yielded excellent early construct validity evidence for the 2 subscales of the tool. Converging evidence from the correlations with health literacy and the known-groups differences in technical readiness and quality-of-care concerns among the educational level attainment groups suggests that this tool is capturing what it was intended to capture, namely, how ready a patient might be for using various digital health services. As might be expected, patients with higher health literacy scores had higher readiness scores on both subscales. Patients with higher educational attainment, as well as younger patients, also had higher readiness scores on technical readiness. Understanding which patients are least digitally ready is the first step toward health equity and is one of the values of the newly designed tool.

The tool encompasses 2 primary domains—technical readiness and quality-of-care concerns—in which patients may experience barriers to digital health readiness. The quality-of-care concerns domain is a unique addition of this screener to the literature, as it moves beyond the more functional questions of digital skills and literacy to explore other barriers based on patients’ experience and perceptions about health care. Specifically, inclusion of quality-of-care concerns differentiates the tool from the recent Digital Health Readiness Questionnaire that was published by a team in Belgium, which included domains of digital use, digital skills, digital literacy, digital health literacy, and digital learnability.^10^

The screening tool in its entirety exemplifies the complexity of factors influencing digital health uptake among various populations and highlights several areas for potential intervention to support increased digital health uptake at both the individual and population levels. Proactive and routine screening for digital health readiness is critical to understand the multifaceted problems leading to inequities in digital health uptake. Use of the tool may allow communities and health systems at a localized level to identify and address the digital health readiness needs of the population they serve. For example, patients who indicate that they are not confident using the internet or do not know how to download a health app could be assigned a digital health navigator to assist with these tasks or a patient who reveals that they do not have access to the internet could be connected to free community-based or hospital-based internet programs. On a national scale, screening for digital health readiness will enable researchers to understand the public health needs related to digital health uptake and policymakers to make solutions to address the root causes of digital health inequities.

### Limitations

There are limitations to this study. The digital screening tool was developed at a single health care institution in an urban setting, which may limit its generalizability. To address this, we strove to enroll a diverse set of participants across each phase of work, with attention to ensuring variable patient demographic characteristics and enrollment across different clinical sites as well as in the community. In addition, most participants scored with higher digital health readiness, which may have affected the ultimate tool by impacting the ultimate priority assigned across subdomains. This is likely the reason for generally high technical readiness and low quality-of-care concerns scores. We also limited enrollment to English-speaking participants and may see additional concepts arise among individuals who do not speak English. Future work administering the revised tool across a wider range of geographic and urban vs rural settings and among individuals who do not speak English may reveal more variability in digital health readiness across these populations. In addition, there were 2 items related to privacy and security that loaded on both factors. We included these 2 items in the quality-of-care concerns composite score as the factor loadings on the quality-of-care concerns factor were twice as large as those on the technical readiness factor (Table 3) and because we felt the content validity of the quality-of-care concerns scale could be improved by including them. As such, the items may reflect some degree of technical readiness as well. However, quality-of-care concerns internal consistency was good for this phase in development, and future work will focus on refining these items. In addition, the technical readiness subscale is quite long at 18 items. Future work will focus on shortening the scale, but not at the expense of content validity, construct validity, or reliability.

## Conclusions

In this qualitative study, we developed a digital health readiness tool to quantify an individual’s digital health readiness and facilitate provision of personalized digital health support to address specific barriers to readiness. Data collected from the screener may aid health care professionals in clinical decision-making and, on a larger scale, may help local organizations, researchers, and policymakers to create solutions. Ultimately, solutions to address digital health inequities cannot be designed without a comprehensive understanding of the barriers individuals face when accessing, understanding, or using digital health tools. Future work is needed to design interventions to support patients identified as having specific digital health readiness barriers and inform development of a shorter version of the tool for routine implementation across clinical care sites.

## Notes

Supplement 1. **eAppendix 1.** Interview Guide for Patients **eAppendix 2.** Interview Guide for Clinicians/Telehealth Support

Supplement 2.
Data Sharing Statement
