# Supplementary material for: Development and Preliminary Validation of a Screener for Digital Health Readiness
Source: JAMA Netw Open. 2024 Sep 10;7(9):e2432718. doi: 10.1001/jamanetworkopen.2024.32718 (PMC11388026; doi:10.1001/jamanetworkopen.2024.32718)
Supplement: Supplement 1. — eAppendix 1. Interview Guide for Patients eAppendix 2. Interview Guide for Clinicians/Telehealth Support [file jamanetwopen-e2432718-s001.pdf]

Supplementary Online Content

Rising KL, Guth A, Gentsch AT, et al. Development and preliminary validation of a screener for digital health readiness. *JAMA Netw Open*. 2024;7(9):e2432718. doi:10.1001/jamanetworkopen.2024.32718

eAppendix 1. Interview Guide for Patients

eAppendix 2. Interview Guide for Clinicians/Telehealth Support

This supplementary material has been provided by the authors to give readers additional information about their work.

© 2024 Rising KL et al. *JAMA Network Open.*

eAppendix 1: Interview Guide for Patients

1. To begin, let's list or discuss challenges you – or people you may know – experience or have experienced when trying to have a video visit with a healthcare provider.
    a. *If needed, ask about things such as:*
        i. *Getting video/audio to work*
        ii. *Not having a stable Wi-Fi connection*
        iii. *Downloading a telehealth application or accessing a telehealth website*
        iv. *Needing a password for a telehealth application or telehealth website*
        v. *Logging into a telehealth application or telehealth website*
2. Thinking about yourself as well as anyone else you know, can you list all the reasons that you can think of for why a person would decide not to have a telehealth visit?
3. Now imagine that a person is interested in having a telehealth visit but has never had one before - can you list all of the reasons that you can think of that you think a person may have problems connecting to a telehealth visit?
4. *(Skip to 7 if they have never had a telehealth appointment before)* When you are able to connect with your healthcare provider, are you able to communicate clearly?
    a. Can you see the provider?
    b. Can you hear the provider?
    c. Were there any problems on either your side or the provider's side? If so, what were they?
5. Do you do video visits alone or does someone help you?
    a. If someone helps you, what part do they help you with?
6. When you face problems – such as WiFi connection or your camera not working – how do you fix it?
    a. Do you have the capability to fix it yourself?
        i. If not, is there someone you can get help from, such as calling your service provider, like Comcast, or involving a friend or family member?
7. Knowing you have not had a telehealth visit before, have you ever been offered a telehealth visit by one of your healthcare providers?
    a. If so, what are the reasons you have never participated in a telehealth appointment?
    b. What would help make you more comfortable or open to having a telehealth appointment?
8. Are there any other challenges we have not covered that you experience when you try to use technology for your health care needs?

Now, let's talk about how you feel about technology in your health care.

9. How much do you trust your provider(s) when you have a video visit with them? In other words, does your trust in your provider change when you have a video visit versus an in-person visit? If so, why?
10. How much do you trust technology to keep your medical information safe?
11. How do you feel about the quality of telehealth visits?
12. How well do you think you can communicate during your telehealth visits?

© 2024 Rising KL et al. *JAMA Network Open.*

13. In your opinion, what would make up a “good” telehealth experience with a healthcare provider? Meaning, what sorts of things would make you want to have another telehealth visit with that provider?
14. When do you think it would be more helpful to have a telehealth visit as opposed to going in-person?
15. With what we have discussed in mind, what education and training would help you better use telehealth resources?

© 2024 Rising KL et al. *JAMA Network Open.*

Appendix 2: Interview Guide for Clinicians/Telehealth Support

1. How often do your patients express not having access to technology?
    a. If they don't have needed technology, what services or assistance do you offer them?
2. Tell us about challenges that your patients have with not knowing how to use technology for telehealth purposes?
    a. When have you seen patients have the greatest challenges? (examples if needed: finding the website/app to connect to the video visits, trouble logging on, audio/visual challenges…)
3. Tell us about any concerns that your patients have expressed about not trusting telehealth resources to keep their information private and safe?
4. Have any of your patients not found telehealth to be an acceptable form of care delivery? Meaning, do they feel as though they are not getting the same level of care that they would receive in-person? If so, please elaborate.
5. For what types of visits do you, as the provider, encourage your patients to do via telehealth versus ones that you prefer to do in-person?
   How do you decide who to offer a telehealth visit to?
6. If you had a group of patients in front of you, and you had to figure out which ones could do telehealth and which ones couldn't, and you could only ask three questions to differentiate the users from the non-users, what would you ask them?
7. How do you provide assistance to patients who are not able to connect to you via a telehealth visit?

© 2024 Rising KL et al. *JAMA Network Open.*
