# Supplementary material for: Development and Preliminary Validation of a Screener for Digital Health Readiness
Source: JAMA Netw Open. 2024 Sep 10;7(9):e2432718. doi: 10.1001/jamanetworkopen.2024.32718 (PMC11388026; doi:10.1001/jamanetworkopen.2024.32718)
Supplement: Supplement 2. — Data Sharing Statement [file jamanetwopen-e2432718-s002.pdf]

Data Sharing Statement

Rising. Development and Preliminary Validation of a Screener for Digital Health Readiness. *JAMA Netw Open*. Published September 10, 2024. doi:10.1001/jamanetworkopen.2024.32718

Data
Data available: No
